# Treatment with ensitrelvir for COVID-19 in hospitalized patients of very advanced age: Case series

**DOI:** 10.1097/MD.0000000000039080

**Published:** 2024-07-26

**Authors:** Takafumi Tomita, Shogo Miyazawa, Takuhiro Sonoyama

**Affiliations:** a Tomita Hospital, Wakayama, Japan; b Shionogi & Co., Ltd., Osaka, Japan.

**Keywords:** advanced age, aged 80 and over, COVID-19, ensitrelvir, SARS-CoV-2

## Abstract

**Introduction:**

Ensitrelvir fumaric acid (ensitrelvir) is an orally active 3C-like protease inhibitor used to treat severe acute respiratory syndrome coronavirus 2 infection. Ensitrelvir was granted an emergency use authorization in Japan in 2022, but reports on the effectiveness and safety of ensitrelvir in actual clinical settings are limited.

**Methods:**

Here, we report a case series of 9 patients with laboratory-confirmed symptomatic coronavirus disease of 2019 (COVID-19) who completed a 5-day course of ensitrelvir at Tomita Hospital from November 2022 to April 2023. Data on clinical symptoms, oxygen saturation, and food intake were collected for 14 days, beginning on the first day of ensitrelvir administration. The outcome of COVID-19 in each patient was also evaluated during this period.

**Results:**

All patients were female, 80 years old or older, and the mean age was 90.2 ± 5.5 years. All patients received ensitrelvir within 2 days after the onset of COVID-19. At baseline, 7 among the 9 patients had their body temperature above 37.5 °C and all of them had oxygen saturation levels of 94% or higher. All patients recovered without worsening of COVID-19, and none received oxygen or additional antiviral drugs during the observation period; no deaths were reported within 14 days. After receiving ensitrelvir for 5 days, all patients had resolution of fever (<37 °C). There was no significant decrease in food intake of patients due to COVID-19. All patients maintained oxygen saturation above 93%.

**Conclusion:**

Our results provide information on the real-world usage of ensitrelvir in elderly, hospitalized patients with COVID-19, and suggests that ensitrelvir is an option for treatment of COVID-19 in these population.

## 1. Introduction

Infections caused by the virus named severe acute respiratory syndrome coronavirus 2 (SARS-CoV-2) led to the pandemic of coronavirus disease of 2019 (COVID-19) disease that began in March 2020. As of March 2023, approximately 33 million SARS-CoV-2 infections and more than 70,000 deaths due to COVID-19 have been reported in Japan.^[1]^ Infections caused by the Omicron variant of SARS-CoV-2 are associated with an increased likelihood of common cold-like symptoms, such as nasal discharge, nasal obstruction, and sore throat.^[2]^ The median incubation period for the Omicron SARS-CoV-2 variant is approximately 3 days, and 20% to 40% of infected individuals remain asymptomatic.^[2]^ The pathogenesis of COVID-19 results from an inflammatory response due to viral proliferation,^[3]^ and the disease is highly transmissible in the early stages of onset. Therefore, initiating antiviral treatment soon after SARS-CoV-2 infection is beneficial to suppress viral proliferation, mitigate excessive inflammation and immune response caused by viral infection, and minimize transmission.

In Japan, molnupiravir and nirmatrelvir/ritonavir are the oral antiviral drugs approved for patients with SARS-CoV-2 infection and risk factors for worsening COVID-19.^[3]^ Clinical trials have demonstrated that these agents are effective in preventing the progression of COVID-19 in unvaccinated patients with risk factors for severe illness.^[4,5]^ However, these studies were conducted before Omicron became the predominant strain of SARS-CoV-2. Additionally, vaccinated individuals were excluded from earlier studies.

Ensitrelvir fumaric acid (ensitrelvir) is an oral drug that is used to treat infection caused by SARS-CoV-2. Similar to nirmatrelvir, ensitrelvir inhibits the 3C-like protease of SARS-CoV-2. The recent phase 3 SCORPIO-SR clinical trial demonstrated a significantly shorter time to resolution of symptoms in patients treated with ensitrelvir versus placebo during a period in which Omicron was the predominant circulating strain of SARS-CoV-2.^[6]^ Based on the result of SCORPIO-SR study, ensitrelvir was granted an emergency use authorization in Japan for those infected with SARS-CoV-2 regardless of risk factors for severe COVID-19, but evidence in clinical practice is limited,^[7,8]^ especially among patients of very advanced age. Although the severity of COVID-19 has become milder with Omicron variant,^[9]^ it has been reported that advanced age is a risk factor for severe COVID-19 even in the Omicron period.^[10–12]^ Therefore, evidence on clinical efficacy of ensitrelvir for people of advanced age is important.

We conducted a retrospective chart review at Tomita Hospital, which provides long-term care for elderly patients requiring convalescent and/or nursing care. Although guidelines recommend the use of molnupiravir or nirmatrelvir/ritonavir for high-risk patients (including older adults) with mild COVID-19,^[2,3]^ ensitrelvir was administered to patients because of difficulties in obtaining the recommended agents. The aim of the present study was to assess the real-world clinical effectiveness and safety of ensitrelvir in hospitalized patients of very advanced age.

## 2. Material and methods

### 2.1. Study design and patient population

**Figure 1.**
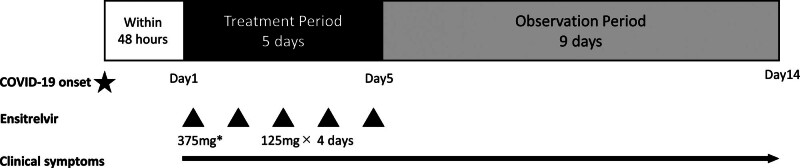
Milestones for case series. All patients were started ensitrelvir treatment within 48 hours of COVID-19 onset. They received oral ensitrelvir 125 mg once daily for 5 days, with the loading dose of 375 mg on the first day. Clinical outcomes including body temperature and food intake were reported for 15 days.

This retrospective chart review was conducted at a single center (Tomita Hospital, Wakayama, Japan) from November 2022 to April 2023. To minimize bias, all patients who received ensitrelvir during the study period were included. Patients received oral ensitrelvir 125 mg once daily for 5 days, with the loading dose of 375 mg on the first day, in accordance with the drug label (Fig. 1). Patients who received ensitrelvir via gastrostomy tube were excluded, as this route of administration differs from that indicated per the drug label.

This study was approved by the MINS Institutional Review Board (MINS-IRB-230219; approved June 21, 2023) and followed the Declaration of Helsinki. Consent of patients was obtained using the opt-out method due to the retrospective nature of this study. This study was registered in the University Hospital Medical Information Network (ID: UMIN000051482; date of first registration: June 30, 2023).

### 2.2. Data collection

Background data collected on each patient included sex, age, underlying diseases/comorbidities, number of doses of COVID-19 vaccine previously received, time from onset of COVID-19 to start of ensitrelvir, and concomitant medications. Clinical symptoms, oxygen saturation (%) and mortality were evaluated for 14 days, including the day of initiation of ensitrelvir treatment (Fig. 1). The outcome of SARS-CoV-2 infection in each patient was assessed by investigators according to clinical symptoms, including body temperature. Given that all patients in the study were 80 years of age or older and required long-term nursing care, it was not feasible to collect subjective symptoms of COVID-19 by interviewing patients. Therefore, each patient’s general condition was assessed based on the amount of food eaten for breakfast, lunch, and dinner. The food intake was quantified using a 10-point scale for the proportion of each main dish and side dish eaten, with a maximum score of 60 points per day.^[13]^

In this descriptive study, continuous variables were reported as means and standard deviations; categorical variables were summarized as frequencies and percentages of the study population. All analysis were performed using R (Ver 4.1.3) and Microsoft Excel Office 365. Missing data were not interpolated. No statistical tests were performed.

## 3. Results

### 3.1. Patient characteristics

Between November 2022 and April 2023, 11 patients at Tomita Hospital received ensitrelvir to treat SARS-CoV-2 infection. Two patients were excluded from the study because they received ensitrelvir via gastrostomy tube (Fig. S1, Supplemental Digital Content, http://links.lww.com/MD/N275). The total population included in this chart review consisted of 9 patients. All patients had laboratory-confirmed symptomatic COVID-19 and their severity was mild at the time of initiation of ensitrelvir treatment.

Background characteristics of the participants are summarized in Table 1. All patients were female and hospitalized. All patients were at least 80 years old, with 4 patients (44.4%) over 90 years of age. All patients received ensitrelvir within 2 days of COVID-19 onset. Only 1 patient was unvaccinated against COVID-19; the other 8 patients had each received 2 or more COVID-19 vaccines.

**Table 1:** Background characteristics.

Variable	Value
Sex	
Female	9 (100.0%)
Age, yrs, mean (SD)	90.2 ± 5.5
*Age categories*	
<80	0 (0.0%)
80–89	5 (55.6%)
90–99	4 (44.4%)
*Patient conditions*	
Hospitalized	9 (100.0%)
*Vaccination, no*.	
0	1 (11.1%)
1	0 (0.0%)
2	1 (11.1%)
3	2 (22.2%)
4	3 (33.3%)
5	2 (22.2%)
*Time from onset to administration*	
<24 hours	8 (88.9%)
≥24 to < 48 hours	1 (11.1%)
*Underlying condition*	
Disuse syndrome	8 (88.9%)
Chronic heart failure	5 (55.6%)
Hypertension	5 (55.6%)
Cerebral infarction	2 (22.2%)
Peripheral neuritis	2 (22.2%)
Deep vein thrombosis	1 (11.1%)
Parkinson disease	1 (11.1%)
Vitamin deficiency	1 (11.1%)
Acute renal failure	1 (11.1%)
Angina pectoris	1 (11.1%)
Thyroid tumor	1 (11.1%)
Uterine fibroid	1 (11.1%)
Dyslipidemia	1 (11.1%)
Severe pressure ulcer	1 (11.1%)
Symptomatic epilepsy	1 (11.1%)
Myocardial infarction	1 (11.1%)
Calculous cholecystitis	1 (11.1%)
Iliopsoas abscess	1 (11.1%)
Iron deficiency anemia	1 (11.1%)
Diabetes mellitus	1 (11.1%)
Refractory reflux esophagitis	1 (11.1%)
Urinary retention	1 (11.1%)
Chronic hepatitis	1 (11.1%)
Chronic renal failure	1 (11.1%)
Chronic cystitis	1 (11.1%)
Bilateral chronic subdural hygroma	1 (11.1%)

Concomitant medications taken by participants are presented in Table S1, Supplemental Digital Content, http://links.lww.com/MD/N274. The most common concomitant medications were laxatives (7/9), antihypertensives (4/9), and antacids (4/9). Additionally, 3 patients received antiplatelets, 2 patients received antiepileptic agents, and 1 received an antiparkinsonian drug. All concomitant medications were prescribed for underlying diseases.

### 3.2. Outcomes

**Figure 2.**
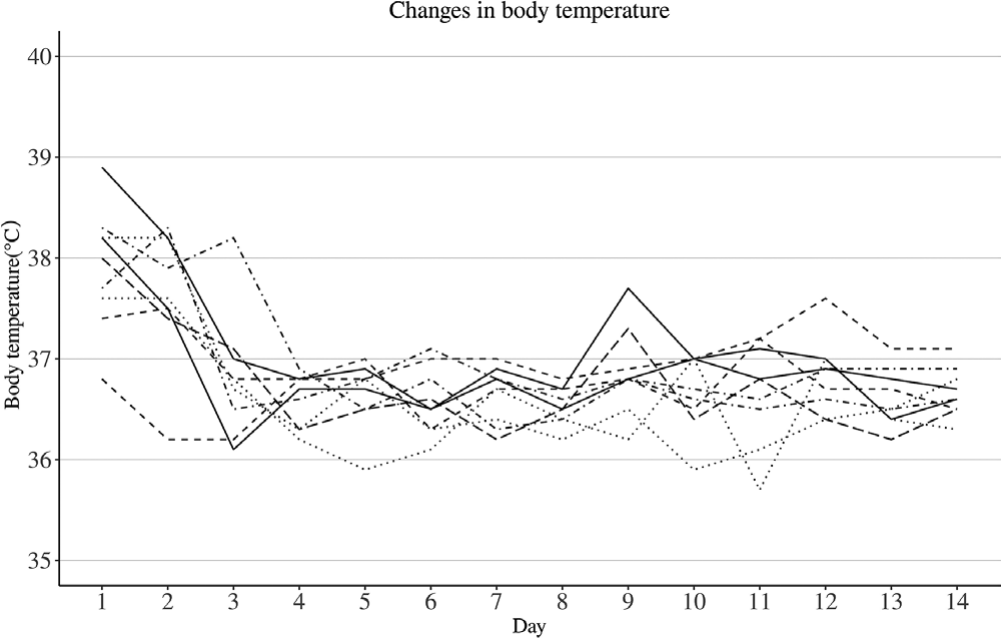
Changes in body temperature by the day. The body temperature of target 9 patients was evaluated. If more than one measurement was performed in a day, the highest value was recorded. Day 1 is the day of the first administration of ensitrelvir.

All patients completed treatment with ensitrelvir and recovered without progression to severe COVID-19. None of the patients required supplementary oxygen or additional treatment with other antivirals. At baseline, 7 among the 9 patients had their body temperature above 37.5 °C. By 4 days after the first administration of ensitrelvir, fever had resolved to <37 °C in all patients; thereafter, although 5 patients experienced a transient rise of the body temperature above 37.5 °C, the temperature was below 37.5 °C at Day 14 in all the 9 patients (Fig. 2). As clinical symptom, 5 among the 9 patients had cough, 2 had sputum and 1 had sweating. Sputum suctioning was performed in the 2 patients with sputum, both of which had recovered by the end of ensitrelvir treatment.

**Figure 3.**
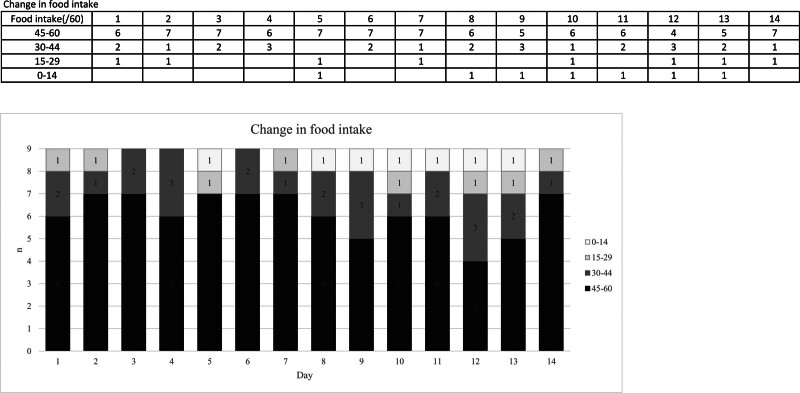
Changes in food intake by the day. The daily food intake of target 9 patients was evaluated on the basis of the amount of food eaten for breakfast, lunch, and dinner, which was quantified using a 10-point scale for the proportion of each main dish and side dish eaten, with a maximum score of 60 points per day. The food intake was categorized as 0 to 14 points, 15 to 29 points, 30 to 44 points and 45 to 60 points. Day 1 is the day of the first administration of ensitrelvir.

A 98-year-old female patient needing total assistance had reduced food intake on Day 5 and Day 8 to Day 13. On Day 5, the amount of food was reduced due to the possibility of aspiration. She vomited on the night of Day 7 for unknown reasons, therefore she was fasted on Day 8 to Day 13 for safety reasons. The other patients did not have significantly decreased food intake during the 14 days after starting on ensitrelvir (Fig. 3).

At baseline, the oxygen saturation levels of the 9 patients were between 95% and 96%. During the course of the treatment and follow up until Day 14, all patients maintained oxygen saturation levels of 93% or higher (Fig. S2, Supplemental Digital Content, http://links.lww.com/MD/N276).

No adverse events related to ensitrelvir were reported, and no patients discontinued the drug. There were no cases who died during the follow-up until Day 14.

## 4. Discussion

This study reported the outcome of ensitrelvir treatment for SARS-CoV-2 among patients older than 80 years of age who are hospitalized in a long-term nursing care facility. The risk of severe outcomes of COVID-19 is reported to increase markedly with increasing age.^[10–12]^ According to a recent report about from China in the Omicron period, patients older than 80 years of age were reported to be 3 times more likely to develop severe diseases than those younger than 70 years of age, with the risk of severe diseases of 8.37% for unvaccinated patients and 2.44% for vaccinated patients.^[10]^ All patients in the study recovered without worsening COVID-19 and without receiving oxygen or other antiviral drugs. Therefore, the results of this study suggest that ensitrelvir is an effective treatment for SARS-CoV-2 infection in hospitalized patients of very advanced age. Furthermore, all study participants completed 5 days of ensitrelvir treatment without any adverse events, indicating that ensitrelvir was safe and well tolerated in these patients.

Since all patients in this study were resident in the nursing hospital and most of them were likely hospital acquired infection, they were able to receive ensitrelvir within 2 days after the onset of COVID-19. Additionally, the hospital`s capability to detect SARS-CoV-2 infections through routine testing also facilitated the timely administration of ensitrelvir. The early administration of ensitrelvir from the onset could contribute to the favorable outcomes.

A limitation of the study is its design as a single-center review that included a small number of patients and no controls. Also, given that all patients in the study had mild COVID-19, the effect of ensitrelvir in very elderly patients with moderate or severe COVID-19 remains unclear. Another limitation of the study pertains to the inability to obtain self-reported data on subjective symptoms of COVID-19 in this population of patients aged 80 years or older. Dietary intake, which was used as a proxy for general acute health and symptom severity, may not necessarily reflect the overall health status of patients.

In this study of hospitalized patients over 80 years of age who received ensitrelvir as treatment for SARS-CoV-2 infection, all participants experienced improved symptoms without worsening of COVID-19 and without evidence of new safety signals related to the drug. Ensitrelvir may be an effective treatment option in older hospitalized adults, including patients older than 80 years of age who are receiving long-term nursing care.

## Acknowledgments

The authors would like to thank the patients and their families. Editorial assistance in the preparation of this article was provided by Tatsuhiro Uenishi of Medilead, Inc., and support for this assistance was funded by Shionogi & Co., Ltd. Tomita Hospital used SIMPRESEARCH® as a data tabulation tool, and we would like to thank the SIMPRESEARCH® developer, 4DIN Ltd. The authors would also like to thank Kentaro Shibata and Yasuko Ariwa for their contribution to manuscript development.

## Author contributions

**Conceptualization:** Takafumi Tomita, Shogo Miyazawa, Takuhiro Sonoyama.

**Data curation:** Shogo Miyazawa.

**Investigation:** Takafumi Tomita.

**Methodology:** Takafumi Tomita, Shogo Miyazawa, Takuhiro Sonoyama.

**Project administration:** Takuhiro Sonoyama.

**Resources:** Takuhiro Sonoyama.

**Writing – review & editing:** Takafumi Tomita, Shogo Miyazawa, Takuhiro Sonoyama.

**Writing – original draft:** Takuhiro Sonoyama.

## Supplementary Material
