# Supplementary material for: Treatment with ensitrelvir for COVID-19 in hospitalized patients of very advanced age: Case series
Source: Medicine (Baltimore). 2024 Jul 26;103(30):e39080. doi: 10.1097/MD.0000000000039080 (PMC11272380; doi:10.1097/MD.0000000000039080)
Supplement: Supplementary file 2 [file medi-103-e39080-s002.docx]

**Supplementary Table 1** Concomitant medications（Day1-Day5）

| **Medication** | **n (%)** |
| --- | --- |
| Preparations for constipation | 7(77.8%) |
| Bisacodyl | 3(33.3%) |
| Senna Extract | 3(33.3%) |
| Sodium picosulfate hydrate | 2(22.2%) |
| Elobixibat hydrate | 1(11.1%) |
| Glycerin | 1(11.1%) |
| Antacid, Anti-ulcer agent | 4(44.4%) |
| Lansoprazole | 3(33.3%) |
| Rebamipide | 1(11.1%) |
| Antihypertensive drug | 4(44.4%) |
| Amlodipine besilate | 3(33.3%) |
| Valsartan | 1(11.1%) |
| Urapidil | 1(11.1%) |
| Anti platelet agents | 3(33.3%) |
| Aspirin | 2(22.2%) |
| Edoxaban tosilate hydrate | 1(11.1%) |
| Prasugrel hydrochloride | 1(11.1%) |
| Anticonvulsant | 2(22.2%) |
| Levetiracetam | 1(11.1%) |
| Lacosamide | 1(11.1%) |
| Antiparkinson drug | 1(11.1%) |
| Levodopa, carbidopa hydrate | 1(11.1%) |
| Antipyretic analgesics | 1(11.1%) |
| Acetaminophen | 1(11.1%) |
| Alzheimer’s drug | 1(11.1%) |
| Donepezil hydrochloride | 1(11.1%) |
