# Supplementary material for: Treatment with ensitrelvir for COVID-19 in hospitalized patients of very advanced age: Case series
Source: Medicine (Baltimore). 2024 Jul 26;103(30):e39080. doi: 10.1097/MD.0000000000039080 (PMC11272380; doi:10.1097/MD.0000000000039080)

**Supplemental Fig. 2.** Changes in oxygen saturation by the day

The oxygen saturation of target 9 patients was evaluated. If more than one measurement was performed in a day, the lowest value was recorded. Day 1 is the day of the first administration of ensitrelvir.


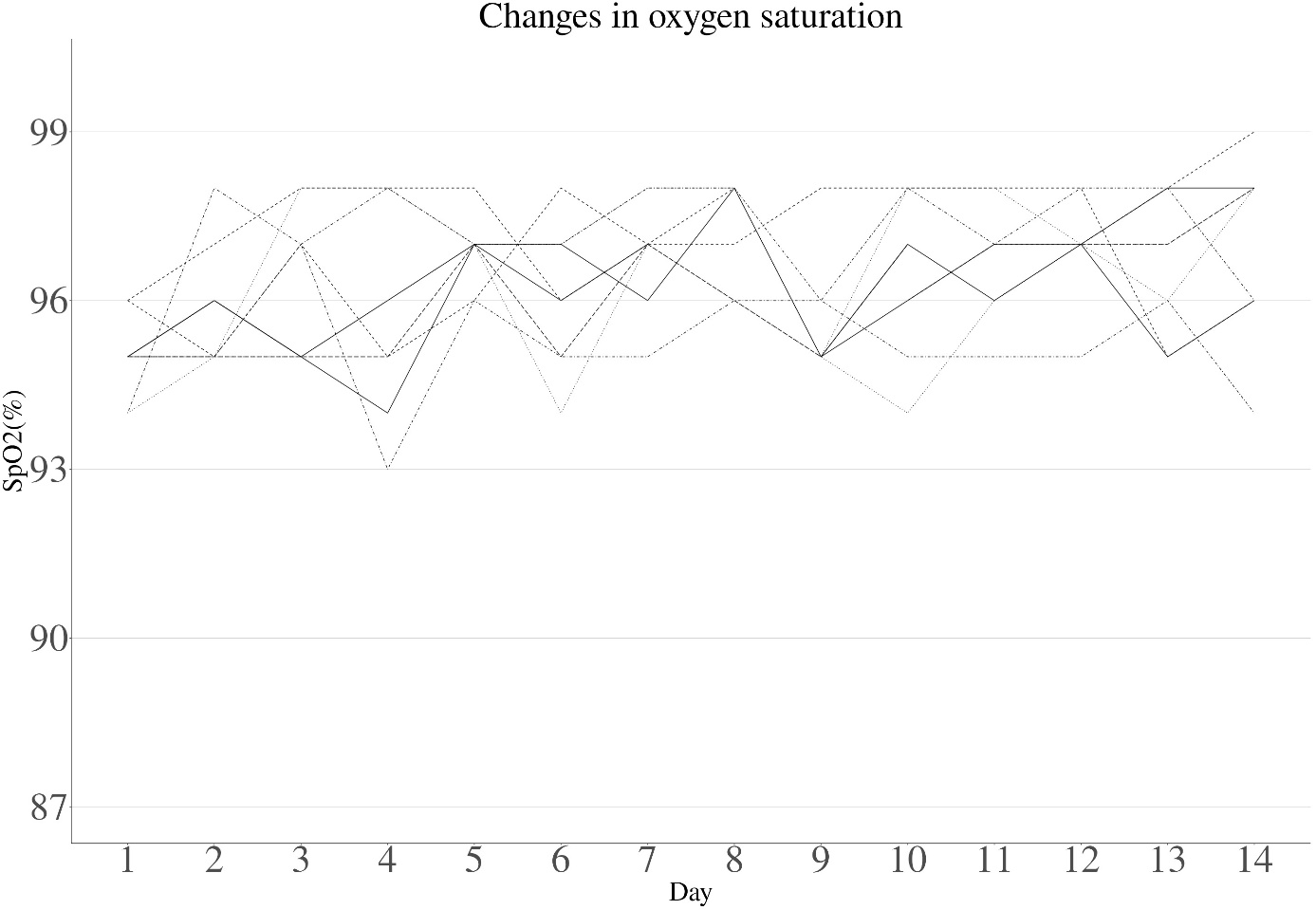

Supplement: Supplementary file 3 [file medi-103-e39080-s003.docx]
